# Parental Reports on Late Effects and Follow-Up Needs: A Single-Center Assessment of Childhood Cancer Survivorship Care in Kenya

**DOI:** 10.3390/curroncol32030162

**Published:** 2025-03-12

**Authors:** Susan Nyabate Mageto, Jesse P. M. Lemmen, Festus Muigai Njuguna, Nancy Midiwo, Sandra Cheptoo Langat, Terry Allan Vik, Gertjan J. L. Kaspers

**Affiliations:** 1 Emma Children’s Hospital, Amsterdam University Medical Center, Vrije Universiteit, Pediatric Oncology, 1105 AZ Amsterdam, The Netherlands; j.lemmen@amsterdamumc.nl (J.P.M.L.); g.j.l.kaspers@prinsesmaximacentrum.nl (G.J.L.K.); 2 Academic Model Providing Access to Healthcare (AMPATH), Moi Teaching and Referral Hospital, Eldoret 30100, Kenya; muigaifes2000@gmail.com (F.M.N.); nancymids1@gmail.com (N.M.); sandralangat@gmail.com (S.C.L.); 3 Department of Pharmacology, School of Health Sciences, Kisii University, Kisii 40200, Kenya; 4 Princess Máxima Center for Pediatric Oncology, 3584 CS Utrecht, The Netherlands; 5 Department of Child Health and Paediatrics, Moi University, Eldoret 30100, Kenya; 6 Department of Pediatrics, Indiana University School of Medicine, Indianapolis, IN 46202, USA; tvik@iu.edu

**Keywords:** childhood cancer, late effects, follow-up

## Abstract

The WHO Global Initiative for Childhood Cancer will likely increase the number of childhood cancer survivors in resource-poor countries. This study explored survivorship care in Kenya through parental reports on late effects and the follow-up needs of childhood cancer survivors. Parents of Kenyan childhood cancer survivors (under 18 years old) who completed treatment for at least one year were interviewed using semi-structured questionnaires from 2021 to 2022. Parents of 54 survivors were interviewed. Survivors had solid tumors (52%) and hematological tumors (48%). Most (52%) received chemotherapy combined with either surgery or radiotherapy. Many survivors (72%) experienced symptoms according to their parents. The most prevalent symptoms were pain (37%), fatigue (26%), and ocular problems (26%). Eleven percent of parents observed limitations in the daily activities of the survivors. Parents of survivors with two or more symptoms were more likely to rate symptoms as moderate to severe (*p* = 0.016). Parents expressed concern about late effects (48%). Only 28% were informed about late effects at the hospital, despite 87% indicating they would have welcomed this information. Follow-up care was deemed important by 98%. Recommendations included providing education about late effects and organizing survivor meetings. Survivorship clinics should be established to ensure that follow-up information and care are accessible.

## 1. Introduction

Interest in pediatric oncology survivorship has been increasing worldwide. Over the years, advancements in childhood cancer diagnosis and treatment have led to improved survival rates. In high-income countries (HICs), survival rates have increased from 30% to 80% [1]. However, 90% of all children with cancer live in low- and middle-income countries (LMICs) where survival rates are still low, with some settings reporting less than 10% survival [2,3].

In 2018, the World Health Organization (WHO) launched a worldwide initiative to improve access to quality care for children with cancer. The initiative aims to improve the overall survival of children with curable types of cancer to 60% by 2030 [4]. The initiative supports governments in building sustainable pediatric oncology programs and centers of excellence. The net effect of this initiative will be more survivors in need of after-treatment care in LMICs.

In Kenya, the focus is gradually advancing from treatment to aftercare, although healthcare providers are still dealing with issues like treatment abandonment and a scarcity of critical medications and supportive care on a daily basis [5,6]. Survivors are reviewed at the same clinic as patients with acute disease or benign hematological conditions. Follow-up reviews usually concentrate on screening for relapses. Moreover, despite an estimated survival rate of 32%, the vast majority of survivors do not adhere to scheduled follow-up appointments beyond one or two years after treatment completion [7].

However, we know that many cancer survivors experience health conditions due to cancer or its treatment [8]. These can include neurological and cognitive impairments, along with psychological and economic challenges, that may hinder the ability to perform everyday tasks [9]. Data on the prevalence of these long-term effects in pediatric oncology survivors in Sub-Saharan Africa are limited. Therefore, survivorship-centered services and research should be established to take care of this advancing population in the region.

This study aimed to assess the symptom burden and follow-up needs of childhood cancer survivors in Kenya through parental proxy reports. Insights into the late effects of childhood cancer treatment and recommendations to strengthen follow-up care would help to shape survivorship programs in LMIC settings like Kenya.

## 2. Materials and Methods

### 2.1. Setting

Kenya is an East African country with a population of close to 53 million. Approximately 39 percent of this population comprises children under the age of 15 [10].

This study was conducted at Moi Teaching and Referral Hospital (MTRH). MTRH is a tertiary-care referral hospital serving the western region of Kenya, which has a population of approximately 24 million [11]. It has a bed capacity of 2000, with 35 allocated to the pediatric oncology department. MTRH receives 250–300 new pediatric oncology patients annually. Acute lymphoblastic leukemia (ALL), non-Hodgkin lymphoma (NHL), and Wilms Tumor comprise almost 60% of the diagnoses made [7]. The pediatric oncology unit is run by 4 doctors and 19 nurses. Treatment options include chemotherapy, surgery, and radiotherapy. In Western Kenya, comprehensive, multimodal pediatric oncology services are exclusively offered at MTRH. Follow-up for childhood cancer survivors in peripheral facilities is not formalized.

Parents of pediatric oncology patients at MTRH can pay with cash or through the Social Health Insurance Fund (SHIF) for medical services, including diagnostics, chemotherapy, supportive care, surgery, and radiotherapy, as well as follow-up care after treatment completion [12,13].

### 2.2. Study Design

This mixed-methods cross-sectional study evaluated the experiences of late-effect symptoms reported by parents and conducted a follow-up of childhood cancer survivors using a semi-structured questionnaire. Parents of childhood cancer survivors (under 18 years at the time of the study) who were newly diagnosed with a malignancy between 1 January 2010 and 31 December 2019 and who had at least one year of event-free survival after completing treatment were invited to participate. By definition, “event-free survival” refers to the absence of any treatment failure: treatment abandonment, progressive or relapsed disease, and death. Between October 2021 and September 2022, one or two interviewers interviewed parents at home or the hospital. Each interview lasted 60–90 min. The parents were asked for informed consent when they were encountered at the outpatient clinic or when approached (in cases of valid contacts) to revisit the clinic.

After an extensive literature review, a panel of Kenyan, American, and Dutch doctors designed the questionnaire. It included both open-ended and structured questions that parents could rate on 2–5-point scales (Supplementary File S1).

The questionnaire was available in English and Kiswahili. The original English version was initially translated into Kiswahili and subsequently back-translated to detect any misapprehensions, mistranslations, or imprecisions. A clinician (a medical doctor, nurse, or pharmacist) conducted the parent interview and completed the questionnaire on paper.

The questionnaire explored the following themes: survivor and parent characteristics, childhood cancer treatment, follow-up care, health insurance status, transportation to MTRH, socio-economic circumstances of the family, medical history, late-effect symptoms, performance status, information availability, preferred follow-up after treatment, peer support, and recommendations for guiding survivors. The symptoms identified during the verbal assessment were not further clinically confirmed.

Socio-demographic and clinical characteristics were extracted from hospital records, including sex, date of birth, diagnosis, date of diagnosis, treatment start and end dates, date of last follow-up, treatment modality, health insurance status, and follow-up duration after treatment completion.

A pilot study was conducted on the parents of five children who met our study’s inclusion criteria to test the questionnaire for content, clarity of language, and cultural sensitivity. Based on this pilot test, minor changes were made to the questionnaire to make questions clearer and more culturally appropriate.

The study protocol was approved by MTRH’s Institutional Research and Ethics Committee (FAN: 0004007).

### 2.3. Data Analysis

Data from parent interviews were fed into password-protected Excel sheets and Castor EDC v2024.4.4.1. Data coding and analysis were performed using SPSS 27. Frequency distributions, medians, means, and standard deviations were calculated. Parent-reported outcomes and socio-demographic and clinical characteristics were compared using Chi-square or Fisher’s exact test for dichotomous dependent data and a two-sided independent *t*-test or ANOVA for continuous dependent data. Bonferroni corrections were applied to account for multiple testing. A two-sided *p*-value of less than 0.05 was considered statistically significant.

## 3. Results

### 3.1. Survivor and Parent Characteristics

From January 2010 to December 2019, 1472 children were newly diagnosed with a malignancy at MTRH. During this period, an estimated 450 of these children completed treatment and were in remission without treatment failure [7].

**Figure 1:**
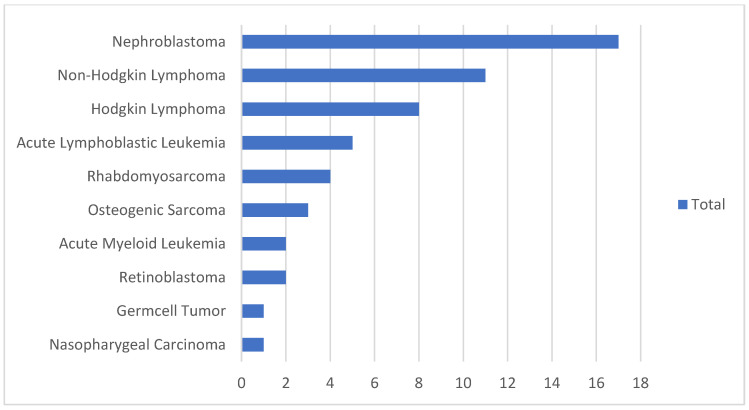
Type of cancer diagnosed in childhood cancer survivors (n = 54).

Valid contact information was available for 60 families. Parents of 54 children were interviewed, either at the hospital (56%) or during home visits (44%). Respondents included mothers (46%), both parents (19%), fathers (11%), grandmothers (4%), or others (20%). Table 1 and Table 2 present the socio-demographic and clinical characteristics of the survivors and their parents. The median age at diagnosis was 5.0 years (IQR 3.0–8.0). A greater number of boys (67%) was represented. Most survivors (52%) had solid tumors (Figure 1).

### 3.2. Childhood Cancer Treatment

Survivors received the following treatment combinations: chemotherapy alone (48%), chemotherapy with surgery (22%), chemotherapy with surgery and radiotherapy (22%), chemotherapy with radiotherapy (6%), and surgery (2%). Among the children prescribed chemotherapy, alkylating agents (91%) and anthracyclines (83%) were included in the treatment regimen. The length of treatment varied from 1 to 26 months, with a median duration of 6.0 months.

### 3.3. Follow-Up

Most survivors (98%) were evaluated during follow-up appointments after treatment completion. The median follow-up duration was 23.0 months (IQR 14.0–36.0). Survivors treated with radiotherapy had a borderline significantly shorter follow-up duration (<23 months versus >23 months; OR 5.5 [Bonferroni-corrected *p* = 0.05; CI 95% 1.2–24.1]) than those treated with chemotherapy only. Twenty-six survivors (48%) were lost to follow-up before they were approached to be enrolled in the study.

### 3.4. Transportation to MTRH

Most survivors traveled over 100 km (65%), used public transport (87%), and took over 3 h (56%) to get to MTRH. Traveling to the hospital was considered expensive (82%), time-consuming (74%), and difficult (65%).

### 3.5. Medical History

The parents of 24% of survivors reported having relatives with cancer. In the six months leading up to the interviews, 37% of survivors had used some form of medication. Antimalarials were the most commonly used (15%), followed by analgesics (5%) and antibiotics (5%). Parents noted that some survivors (13%) were consulting a doctor for various conditions, including allergies, asthma, headaches, vomiting, eye discharge, HIV, hypertension, limb weakness, and maxillofacial issues.

### 3.6. Parent-Reported Symptoms

Table 3 provides an overview of symptoms reported by parents of childhood cancer survivors. Parents of 39 survivors (72%) noted 14 different types of symptoms in their children (Table 3). The most commonly reported symptoms were pain (37%), fatigue (26%), and ophthalmological issues (26%). Six percent experienced psychological and cognitive challenges. The number of late-effect symptoms reported by parents per survivor ranged from zero to nine, with a median of two. The severity of symptoms was classified as mild (47%), moderate (33%), or severe (19%). Parents of survivors with two or more reported symptoms rated their child’s symptoms as moderate to severe more often (64%) than those with only one symptom (12%; *p* = 0.016). In girls, symptoms were more frequently rated as mild compared to boys (77% versus 30%; *p* = 0.014).

Pain (37%) was described by parents as recurrent (80%) or long-lasting (20%). Headache was the most common source of pain, followed by abdominal pain; joint pain and jaw pain were also reported by parents. They observed that their children experienced fatigue (26%) more quickly than other children when walking, playing, working, and engaging in strenuous activities. Parent-reported ophthalmological issues (26%) included eye redness, tearing, and itchiness. Ear, nose, and throat problems (15%) comprised tinnitus, hearing loss, postnasal drip, vertigo, nosebleeds, and tonsillitis. Dental issues (11%) involved mouth sores, tooth decay, bad odor, teeth discoloration, and jaw problems. Only one survivor underwent amputation. One survivor required a hearing aid. Cardiac issues (6%) included awareness of heartbeat and hypertension. Psychological challenges (6%) encompassed depression, anxiety, behavioral changes, and loneliness. Cognitive difficulties (6%) involved memory issues, concentration problems, and slow responses, according to the parents. Only one survivor had consulted a psychological counselor.

Fourteen survivors (26%), comprising four girls and ten boys, had entered puberty by the time of the interview. The parent-reported median age for entering puberty was 13.0 in boys (n = 10) and 15.0 years in girls (n = 3). Four girls had begun their menstrual cycles. Most survivors (56%) were underweight (BMI < 18.5), with a median BMI of 15.9 (IQR 14.5–17.7).

Parents reported that the performance of daily life activities was restricted for 11% of survivors in the last 4 weeks. Survivors faced limitations in school attendance (9%), social activities (6%), personal care (4%), and physical work (4%). Treatment modalities were not associated with an increased number or severity of symptoms or with performance status.

### 3.7. Information About Late Effects at MTRH

Fifteen parents (29%) reported being informed about the late effects of cancer treatment at MTRH. Physical issues (heart injury, fatigue, and infertility) were mentioned more frequently (60%) than mental issues (depression, anxiety, and trauma) and other concerns (e.g., radiotherapy effects), which were at 27% and 40%, respectively. Parents (48%) and their children (15%) expressed worries about these late effects. Many parents (87%) wished they had received more information about these late effects.

### 3.8. Preferred Follow-Up

Almost all parents considered follow-up visits important (98%) and necessary (85%). Parents felt it was best to attend the follow-up clinic after treatment completion within six months (52%), within one year (35%), within 1–5 years (9%), over five years (2%), and never (2%). Parents preferred their children to go for follow-up appointments at MTRH (80%) rather than a regional hospital (20%). Although only 44% of parents had informed their county hospitals (nearest to home) about their child’s cancer history, most parents (74%) wanted the county hospitals to be aware. The reasons some parents (26%) chose not to inform the county hospital included the following: the county hospital’s inability to initially diagnose their child, a preference to continue care at MTRH since treatment began there, and concerns about stigmatization from the local community if they revealed the child’s condition. Some families (15%) avoided going to hospitals due to negative experiences during their child’s cancer treatment.

### 3.9. Peer Support

Most parents (94%) and survivors (83%) expressed a desire to connect with other childhood cancer survivors and their families. Parents noted that only 44% of children discussed cancer with those around them: with their mother (44%), doctor (44%), nurse (43%), siblings (35%), father (32%), friends (32%), teacher (28%), or religious leader (17%).

### 3.10. Recommendations for Guidance of Survivors

The parents offered several suggestions on how survivors could be best supported after completing treatment (Table 4).

## 4. Discussion

We performed a parental assessment of childhood cancer survivor symptoms and explored follow-up needs at the largest tertiary hospital in Western Kenya. Many survivors experienced late symptoms after treatment completion, and their parents expressed an urgent wish to continue participating in follow-up care. The 72% prevalence of symptoms among our survivors was higher than what has been reported in studies derived from the US (62%) and South Korea (60%), similar to a Dutch cohort (75%), yet lower than those reported in Canada (92%) [8,14,15]. Symptoms were graded as severe by 19% of parents. In contrast to 72% of parents reporting symptoms in their children, only 13% had recently consulted a doctor because of health issues. This might stem from parents being unaware of late effects, the costs and time required to visit the clinic, the fact that survivors may not perceive the potential urgency of their symptoms, and families having competing priorities.

Pain was the most frequent symptom reported by parents in this study (37%), with 80% classifying their child’s pain as recurrent and 20% as chronic. Thus, chronic pain in our study accounted for 7% compared to 11–44% reported in previous studies [16,17]. This difference may be due to the types of cancers included in the study. Survivors of brain cancer and those of bone and soft tissue sarcomas are more likely to experience cancer-related pain as a result of radiation and amputation [16]. Overall, headache was the most common type of pain reported, in line with the findings reported by Lu et al. [18]. Fatigue was experienced by 26% of survivors, according to the parents. Reported rates of fatigue have varied considerably across studies (14–30%), whereby variations have partly been attributed to the differences between measurement instruments [19]. We did not subject participants to a validated fatigue scale in this study. Eye problems were noted in 26% of survivors by their parents, a figure that was higher than the documented 3% and 7% in two previous studies [14,20]. Fortunately, parent-reported eye redness, tearing, and itchiness for survivors in our study are very manageable and unlikely to be due to the effects of therapy. Only 6% of our survivors had parent-reported cardiovascular problems (palpitations and hypertension). The absence of cardiotoxicity may be attributed to relatively low anthracycline doses used to treat Kenyan children with cancer. More importantly, heart failure will manifest subclinically and is generally not detected subjectively until the condition has progressed to an advanced stage. The reasons for a lack of endocrine, renal, and respiratory parent-reported symptoms may be similar. However, this contrasts with findings reported from HICs [8,21,22]. We found a low prevalence of psychological and neurocognitive parent-reported symptoms. Traditionally, the scope of survivorship care has been on physical rather than psychosocial late effects. This discrepancy is even more pronounced in LMIC setups [23]. A multidisciplinary approach should be employed in survivorship care to ensure that these health issues are not only identified but also monitored and treated.

The longer the period after diagnosis, the higher the likelihood of developing late effects [24]. Also, survivors who have been treated with radiotherapy or were diagnosed with bone or brain tumors have an increased risk of developing severe late effects [25,26]. In our study, the median follow-up time was only 23 months, 28% of survivors had received radiotherapy, 6% had bone cancers, and none had brain cancer. The overrepresentation of ALL, lymphoma, and nephroblastoma survivors in this study roughly resembles the distribution of diagnoses in patients receiving acute treatment [7]. Of the fifteen survivors who received radiotherapy in our study, ten had a nephroblastoma, which is treated with a relatively low dose of radiotherapy. HICs employ more intense treatment regimens due to better supportive care facilities compared to LMICs. Higher doses are generally avoided in LMICs because of a lack of adequate supportive care in those settings [27].

Our study found no association between patient or treatment characteristics and the presence or number of symptoms or daily life hindrances. The distribution of diagnoses, the use of low-toxicity protocols, and a short follow-up duration may explain the absence of these associations in this study [25,28,29]. We relied on proxy reports for this study due to age restrictions applied to patient interviews at the study center. Parent-reported survivor assessments can circumvent age restrictions when performing survey studies on children to estimate the burden of late effects [30]. A 79% parent-reported late-effect prevalence in Australia and New Zealand corresponds with the findings in our study [31]. Fardell et al. reported that 39% of parents observed a negative impact on their children’s quality of life during follow-up, seemingly higher than the 11% who reported daily limitations in our study [30]. Children may experience a higher burden of functional impairment compared to their parents, suggesting that our results might have differed if children had self-reported their health status [32].

Only 29% of parents recalled being informed about the late effects of cancer treatment at MTRH. Half of all parents were concerned about these late effects on their children. This finding aligns with other studies showing that 49% and 33% of parents express worry about late effects [33]. Most parents in our study wished to receive more information on this topic. This concern is not limited to low- and middle-income countries (LMICs); parents in high-income countries (HICs) also report a strong need for sufficient information on late effects [34]. For instance, a study in America found that childhood cancer survivors and their parents often underestimate the likelihood of developing late effects [35]. This underscores the necessity for proper education for both parents and survivors. Furthermore, information about late effects may boost and sustain follow-up adherence [36].

Almost all survivors in this study were initially followed up after treatment completion. The median follow-up time, 23 months, differed from those in India (9 years), South Korea (8 years), and Thailand (3 years) [14,22,37]. This difference in follow-up times may be due to the lack of a specialized follow-up clinic to support survivors at MTRH, resulting in frequent instances of loss to follow-up. Additionally, costs associated with traveling to MTRH and screening tests may prohibit patients from seeking survivorship care and treatment for late effects. In view of these challenges, non-pharmacological alternatives such as cognitive behavioral therapy and physical exercise activities should be promoted [38]. Interventions incorporating physical activity have been shown to reduce the risk of specific late effects such as fatigue, depression, and cognitive deficits [39]. Text messages or phone interviews have been implemented, also in East Africa, to remind adolescent and young adult survivors about their clinic appointments and to connect them with relevant resources [40,41]. Shared care may also be employed to ensure that childhood cancer survivors who need to cover long distances to an oncology center can access care closer to their homes [42]. The introduction of survivorship care plans in Kenya to inform parents, survivors, and healthcare providers could be another intervention that has proven effective in increasing awareness about late effects in this at-risk population [43]. Finally, advocacy to ensure that follow-up care will be included in the national health insurance scheme should also be pursued [44].

Both survivors and parents expressed a desire to engage with other childhood cancer survivors and their families. Peer contact has been shown to be supportive for both survivors and their families in countries such as the US, the Netherlands, and China [45]. Furthermore, peer groups give members a sense of connection, belonging, and experiential knowledge, as older survivors are able to share their experiences [46]. The parents noted that only 44% of the children in our study discuss cancer with those around them, with mothers being the individuals they talk to most often. Peer groups may enable children to interact with other survivors and exchange their cancer experiences.

The parent-reported symptoms in this study were not compared to a healthy or sibling control group. Consequently, we described our findings as late-effect-like symptoms rather than objectively measured late effects. Additional limitations of this study included a small sample size, selection bias due to the non-random selection of participants, a short follow-up duration, and the parent proxy assessments. All these factors may have contributed to either an over- or underestimation of the prevalence of late effects among childhood cancer survivors and should be accounted for when planning future prospective survivor cohort studies in Kenya [47,48].

## 5. Conclusions

This study showed that the parent-reported symptom burden among childhood cancer survivors in Western Kenya is high, despite the relatively short follow-up period. Establishing survivorship clinics, survivorship care plans, and peer support groups would support the provision of structured information on late effects and survivorship among childhood cancer survivors and their families during and after the completion of treatment.

## Figures and Tables

**Table 1:** Socio-demographic and clinical characteristics of childhood cancer survivors (N = 54).

		N/Median	%/IQR
SURVIVORS			
Age	At diagnosis	5.0	3.0–8.0
Sex	Male	36	67%
	Female	18	33%
Type of cancer	Solid tumor	28	52%
	Hematological tumor	26	48%
Health insurance during treatment	Present	52	96%
	Absent	2	4%
Health insurance during follow-up	Present	48	89%
	Absent	6	11%
Modality of treatment	Chemotherapy	53	98%
	Surgery	25	46%
	Radiotherapy	15	28%
Duration of treatment (n = 53)	<6 months	24	45%
	≥6 months	29	55%
Duration of follow-up *	<1 year	11	20%
	1–<3 years	29	54%
	3–5 years	9	17%
	>5 years	5	9%
Follow-up status **	In follow-up	28	52%
	Lost to follow-up	26	48%
School attendance	Primary school	44	81%
	High school	10	19%

* Follow-up duration is defined as time between treatment completion and last hospital visit. ** Follow-up status defines survivors as being “in follow-up” if the last hospital visit took place <24 months prior to interview. Survivor is “lost to follow-up” if last hospital visit was ≥24 months prior to interview.

**Table 2:** Socio-demographic and clinical characteristics of childhood cancer survivors’ parents (N = 54).

		N/Median	%/IQR
PARENTS			
Age (in years)	Father (n = 42)	45.0	39.0–48.3
	Mother (n = 53)	39.0	32.0–43.0
Marital status (n = 53)	Married	38	(72%)
	Divorced/separated	7	(13%)
	Single	5	(9%)
	Widowed	3	(6%)
Number of children in family	Median	4.0	3.0–5.0
	Median (range)	4	(1–11)
Parental education level * (n = 53)	Low	22	42%
	High	31	58%
Religion (n = 53)	Christian	50	93%
	Muslim	3	6%
Distance to MTRH	<50 km	7	13%
	50–100 km	12	22%
	>100 km	35	65%
Travel time to MTRH	<1 h	5	9%
	1–3 h	19	35%
	>3 h	30	56%

* Parental education level classified children as having parents with low or high education. Parents with the highest education level determined the assigned level. Parents without education or attending primary school were defined as having a low education level. Parents attending high school or tertiary education were classified as having a high education level.

**Table 3:** Overview of type and severity of late effects in childhood cancer survivors reported by their parents (n = 54).

Late Effects			Severity					
	Overall Frequency		Mild		Moderate		Severe	
	*n*	%	*n*	%	*n*	%	*n*	%
Pain	20	37%	13	65%	4	20%	3	15%
Fatigue	14	26%	7	50%	5	36%	2	14%
Ophthalmological problems	14	26%	9	64%	3	21%	2	14%
Gastrointestinal	12	22%	8	67%	3	25%	1	8%
Shorter stature than siblings	10	19%	-	-	-	-	-	-
Ear/nose/throat problems *	8	15%	0	0%	5	71%	2	29%
Orthopedic problems *	8	15%	5	71%	2	29%	0	0%
Dental problems	6	11%	3	50%	3	50%	0	0%
Hearing loss	4	7%	-	-	-	-	-	-
Psychological problems *	3	6%	0	0%	2	100%	0	0%
Cognitive problems	3	6%	0	0%	3	100%	0	0%
Cardiac problems	3	6%	0	0%	3	100%	0	0%
Other problems	2	4%	1	50%	1	50%	0	0%
Neurological problems	1	2%	0	0%	1	100%	0	0%
Endocrine problems	0	0	0	0%	0	0%	0	0%
Respiratory problems	0	0	0	0%	0	0%	0	0%
Renal problems	0	0	0	0%	0	0%	0	0%
Secondary malignancy	0	0	0	0%	0	0%	0	0%

* Severity data were missing for one patient.

**Table 4:** Recommendations for guidance of survivors.

1. Education on the late effects of cancer treatment should be provided to both parents and survivors.
2. Healthcare providers should conduct home visits with survivors.
3. Regular phone calls to check on survivors and families.
4. Organize survivorship meetings.
5. Offer psychological assistance to survivors and their families.
6. Provide financial support to parents and survivors, especially for those who travel long distances.
7. MTRH should improve file retrieval during follow-up clinics.
8. MTRH should provide more frequent follow-up clinics.

## Data Availability

The raw data supporting the conclusions of this article will be made available by the authors upon request.

## Supplementary Materials

The following supporting information can be downloaded at https://www.mdpi.com/article/10.3390/curroncol32030162/s1, File S1. Parent Questionnaire.

## Abbreviations

The following abbreviations are used in this manuscript:

LMICs	Low- and Middle-Income Countries
NHIF	National Health Insurance Fund
WHO	World Health Organization
HICs	High-Income Countries
