# Supplementary material for: Parental Reports on Late Effects and Follow-Up Needs: A Single-Center Assessment of Childhood Cancer Survivorship Care in Kenya
Source: Curr Oncol. 2025 Mar 12;32(3):162. doi: 10.3390/curroncol32030162 (PMC11941395; doi:10.3390/curroncol32030162)
Supplement: Supplementary file 1 [file curroncol-32-00162-s001.zip › curroncol-3488777-supplementary.pdf]

CONSENT FORM - PARENTS

<u>Participants of study:</u> Parents of childhood cancer survivors who were diagnosed at MTRH between January 2010 and December 2019 and completed treatment.

The study you are about to participate in explores the parents' perspectives on experiences of their children after cancer treatment completion. The insights obtained through this study will help us to improve guidance of children with cancer.

Should you agree to participate in the study, you will be interviewed about your child's experiences after cancer treatment completion at MTRH.

Talking about the condition of your child may be emotional, but also helpful.

All data collected from you will be dealt with confidentially. The interview will only be available to two researchers.

Any additional information about the study results will be provided to you at its conclusion, upon your request.

You are free to withdraw from the study at any time. Should you agree to participate, please sign your name below, indicating that you have read and understood the nature of the study, and that all your inquiries concerning the activities have been answered to your satisfaction.

……………………………………	……………………………………..
Signature of participant and date	Signature of researcher and date
……………………………………	……………………………………
Signature of participant and date	Signature esearcher and date

DATA COLLECTION FORM - PARENTS

Date of birth: Day: Month: Year:
Gender: Male/ female
Study number:

According to Medical Record:

Diagnosis:
Date of diagnosis at MTRH: Day: Month: Year:
Age at diagnosis: Years:

Date of start treatment: Day: Month: Year:
Date of treatment completion: Day: Month: Year:
Date of last follow-up: Day: Month: Year:

Type of treatment: Chemotherapy: yes/ no
If yes, type: Anthracyclines/ Alkylating agents/ Other
If anthracyclines, dosage:
If alkylating agents, dosage:

Surgery: yes/ no
If yes, specify:

Radiotherapy: yes/ no
If yes, localization: head/ neck/ thorax/ abdomen/ extremities/ craniospinal/ total body

According to Parental Interview:

Interview of: *Mother/ father/ both parents/other caregiver (specify):*
Date of interview: *Day: Month: Year:*

Primary caregiver:
1)Who is primary caregiver of child? *Mother/ father/ grandmother/ grandfather/ other (specify):*

Parental background:
2) Background of father: *Known/ unknown*
a) Age:
b) Education: *No education/ Primary school/ High school/ Tertiary education(specify):*
c) Occupation:
d) Religion*:* *Christian/ Muslim/ None/ Other (specify):*

3) Background of mother: *Known/ unknown*
a) Age:
b) Education: *No education/ Primary school/ High school/ Tertiary education(specify):*
c) Occupation:
d) Religion: *Christian/ Muslim/ None/ Other (specify):*

Marital status:
4) What is marital status of the biological parents? *Single/ married/ divorced/ separated/ widowed*

Siblings:
5) How many children do you have? *Alive: Deceased: Total:*

Transportation:
6) Residence (county)…………………

7) Distance from MTRH: <50 km/ 50-100km/ >100 km

8) What main mode of transportation do you use to reach MTRH?
Walking/ Public transport/ Renting a vehicle/ Private motorbike/ Private car/ Other (specify):

9a) What is the travel time to MTRH? *<1 hour/ 1-3 hours/ >3 hours (specify):*
b) Is traveling to hospital : Time consuming: *yes/ no* Expensive: *yes/ no* Difficult: *yes/ no*

Health-insurance status:
10a) Did you have health-insurance (NHIF) during cancer treatment of your child? *Yes/ no*
b) If yes, when did you have active health-insurance during cancer treatment of your child?
At diagnosis/ During treatment
c) Do you currently have health-insurance (NHIF)? *Yes/ no/ do not know*

LATE EFFECTS

Medical history:

1) What type of cancer did your child have?

2) After being treated for first type of cancer, did your child develop another type of cancer? *Yes/ no*
If yes, please specify which type:

3) Are there other people in the family who have had cancer? *Yes/ no*
If yes, please specify: Father/ mother/ sibling/ other (please specify):
If yes, please specify type of cancer:

4) Has your child used any medication in the last 6 months? *yes/ no*
If yes, which medicines?

5) Does your child have diseases that he/she consults a doctor for right now? *yes/ no*
If yes, which diseases?

School:

1a) Does your child go to school? *yes/ no*
b) What is the highest school level your child attended?
No education/ Primary school/ High school/ Tertiary education(specify):

Performance:

1) How often was your child restricted in personal care (shower, dressing, toilet) during the last 4 weeks due to his/her physical condition?
never/ sometimes/ often/ always

2) How often was your child restricted in <u>physical work</u> (farming, construction, driving, cleaning) during the last 4 weeks due to his/her physical condition?
never/ sometimes/ often/ always

3) How often was your child restricted in <u>social activities</u> (family visits, church visits, holidays, sports) during the last 4 weeks due to his/her physical condition?
never/ sometimes/ often/ always

4) How often was your child restricted in <u>daily chores</u> (groceries, preparing dinner, homework) during the last 4 weeks due to his/her physical condition?
never/ sometimes/ often/ always

5) How often was your child restricted in <u>school</u> during the last 4 weeks due to his/her physical condition?
never/ sometimes/ often/ always/ not applicable because child does not attend school

<u>Physical complaints:</u>

1) Does your child experience <u>pain</u> in his/her body? *yes/ no*

If yes, please explain:

If yes, is pain: Recurrent/ longstanding

If yes, please specify severity: Mild/ moderate/ severe

2) Does your child get <u>tired</u> faster than other persons of its age? *yes/ no*

If yes, please explain:

If yes, please specify severity: Mild/ moderate/ severe

3) Does your child have <u>heart problems</u>? *yes/ no*

If yes: Pain at chest during exercise/ Pain at chest in rest/ Awareness of heartbeat/ Hypertension/ Angina pectoris/ Myocardial infarction/ Cardiomyopathy/ Other (please specify): **(More than 1 answer is possible)**

If yes, please specify severity: Mild/ moderate/ severe

4) Is your child <u>shorter in length</u> than its siblings? *yes/ no*

5) Has your child been diagnosed with hormonal <u>problems (like diabetes</u>/ growth hormone/ thyroid problems) yes/ no

If yes: Diabetes mellitus/ growth hormone deficiency/ thyroid disorders (hypothyroidism, hyperthyroidism, struma)/ Other (please specify): **(More than 1 answer is possible)**

If yes, please specify severity: Mild/ moderate/ severe

6) Does your child have lung problems? *yes/ no*

If yes: Easily out of breath during exercise/ Three or more lung infections in a year/ Coughing more than 6 weeks a year/ Other (please specify): **(More than 1 answer is possible)**

If yes, please specify severity: Mild/ moderate/ severe

7) Does your child have hearing loss?

If yes: does your child use a hearing aid? *Yes/ no*

8) Does your child have ear, nose and throat problems? (like sinus/ smell/ tinnitus problems) *yes/ no*

If yes: Tinnitus/ Rhinitis/ Sinus infections/ Postnasal drip/ Change in smell/ Other (please specify): **(More than 1 answer is possible)**

If yes, please specify severity: Mild/ moderate/ severe

9) Does your child have eye problems? *yes/ no*

If yes: Dryness/ Tearing/ Sensitivity to light/ Vision loss/ Cataract/ Other (please specify): **(More than 1 answer is possible)**

If yes, please specify severity: Mild/ moderate/ severe

10) Does your child have neurological problems? *yes/ no*

If yes: Numb feeling of limbs/ Sensory loss/ Tingling/ Seizures/ Motor dysfunction/ Hemiparesis/ Paralysis/ TIA/ Stroke/ Other (please specify): **(More than 1 answer is possible)**

If yes, please specify severity: Mild/ moderate/ severe

11) Does your child have kidney problems? *yes/ no*

If yes: Urinary tract infections/ Other (please specify): **(More than 1 answer is possible)**

If yes, please specify severity: Mild/ moderate/ severe

12) Does your child have gastro-enterology problems? *yes/ no*

If yes: Chronic diarrhea/ Cramping / Other (please specify): **(More than 1 answer is possible)**

If yes, please specify severity: Mild/ moderate/ severe

13) Does your child have bone (orthopedic) problems? *yes/ no*

If yes: Amputation/ Scoliosis/ Low back pain/ Joint problems/ Other (please specify): **(More than 1 answer is possible)**

If yes, please specify severity: Mild/ moderate/ severe

14) Does your child have teeth or mouth problems? *yes/ no*
If yes: Dry mouth/ Weird taste/ mouth sores/ infections/ tooth decay/ Other (please specify): **(More than 1 answer is possible)**

If yes, please specify severity: Mild/ moderate/ severe

15) Does your child have psychologic problems? *yes/ no*
If yes: Depression/ Anxiety/ Personality or behavior change/ Psychosis/ Other (please specify): **(More than 1 answer is possible)**

If yes, please specify severity: Mild/ moderate/ severe

16) Does your child have cognitive problems? *yes/ no*
If yes: Concentration problems/ Memory problems/ Other (please specify): **(More than 1 answer is possible)**

If yes, please specify severity: Mild/ moderate/ severe

17) Did your child ever visit a psychologist or psychological counsellor? *yes/ no*

18) Does your child experience other physical or mental problems? *yes/ no*
If yes, please specify:
If yes, please specify severity: Mild/ moderate/ severe

Puberty:

1) Did you child enter puberty? (got armpit and pubic hair) yes/ no
If yes: At what age did your child enter puberty (got armpit and pubic hair)?

IN CASE OF DAUGHTER:
2) Did your daughter already have menses? *yes/ no/ do not know/ not applicable < 10 years*

Information:

1a) Has your family ever been told at MTRH about late effects of cancer treatment? *yes/ no*
1b) Do you know which topics were covered? Yes / no
1c)
If yes, which topics were covered?
- Physical problems (for example heart injury, fatigue, infertility) *yes/ no*
- Mental problems (depression, anxiety, trauma) *yes/ no*
- Other (please specify):

2a) How often does your child worry about late effects of treatment? *never/ sometimes/ often/ always*

b) How often do you worry about late effects of treatment? *never/ sometimes/ often/ always*

3) Would you like to have received more information about late effects of cancer treatment? *yes/ no*

Follow-up:

1) Has your child been attending follow-up clinic after he/she finished treatment? *yes/ no*

2a) Do you believe that attending follow-up clinic after completion of treatment is important? *yes/ no*

b) Do you believe that attending follow-up clinic after completion of treatment is necessary? yes/ no

c) How often after completion of treatment would you like to go for a follow-up in the hospital?

Within half year/ Within a year/ Between 1 and 5 years/ After 5 years/ Never

3) Where would your child like to go for their follow-up clinic after cancer treatment?

MTRH/ Regional hospital/ Traditional healer/ Other (please specify):

4a) Is the county hospital (nearest to home) informed about your child's previous cancer history? *yes/ no*

b) Would you like the county hospital to know about your child's cancer history? *yes/ no*

If no, why not?

5) Do you avoid visiting hospitals, because of the experiences you have had during the cancer treatment of your child? *yes/ no*

Peer support

1a) Has your child ever been in contact with other childhood cancer survivors? *yes/ no*

b) Would your child like to get in contact with other childhood cancer survivors? *yes/ no*

c) Have you ever been in contact with other families of childhood cancer survivors? *yes/ no*

d) Would you like to get in contact with other families of childhood cancer survivors? *yes/ no*

e) If you or your child have met other childhood cancer survivors or their families, where was this?

2a) Does your child talk to people around him/her about the cancer? *yes/ no*

b) With whom does your child talk about the cancer? **(More than 1 answer is possible)**

Mother/ Father/ Siblings/ Friends/ Teacher/ Religious leader/ Traditional healer/ Doctor/ Nurse/ Other (please specify):

3) How often does your child still feel like he/she is a cancer patient? *never/ sometimes/ often/ always*

4) Can you give recommendations how children with cancer can best be guided/ followed-up after finishing treatment?

IF THE CHILD IS PRESENT:

→ Measure weight of child:

→ Measure height of child:

→ Calculate BMI=

→ Classify BMI category: Underweight/ normal weight/ overweight/ obesity
